# Polygenic risk indices for schizophrenia, bipolar and major depressive disorder associated with addiction (smoking and alcohol) in the Russian population in patients with schizophrenia or schizophrenia spectrum disorder

**DOI:** 10.1192/j.eurpsy.2026.11271

**Published:** 2026-07-31

**Authors:** V. E. Golimbet, T. V. Lezheiko, N. V. Kondratyev, E. V. Semina, Y. A. Chaika

**Affiliations:** 1 Clinical genetics laboratory; 2 Mental Health Research Center, Moscow, Russian Federation

## Abstract

**Introduction:**

Tobacco and alcohol use are major risk factors for death and disability, but their impact on mental health remains controversial (Griswold M. G. et al., 2016; World Health Organization (Tobacco, Alcohol), 2022).

**Objectives:**

To study how polygenic risk scores for schizophrenia (SZ-PRS), bipolar disorder (BPD-PRS) and major depressive disorder (MDD-PRS) are associated with tobacco and alcohol use in patients with schizophrenia.

**Methods:**

The sample consisted of 887 patients diagnosed with schizophrenia or schizophrenia spectrum disorders. Information on smoking and alcohol use was obtained from patient records and interviews. The polygenic risk score for schizophrenia SZ-PRS (Trubetskoy V. et al., 2022), bipolar BPD-PRS (Adams, James M. et al., 2025) and major depressive disorder MDD-PRS (Nemani A. et al., 2025) was used as an indicator of genetic predisposition, calculated as a weighted sum of single nucleotide polymorphisms that were associated with mental disorders in a genome-wide association study. For statistical processing of the data, a logistic regression model with stepwise algorithms for inclusion and exclusion of predictors was used. Student’s t-test was used to test the equality of the means in the two samples (gender-dependence, PRS-dependence).

**Results:**

The study showed that higher SZ-PRS/MDD-PRS values were associated with regular smoking (p=0.01), (p=0.06) and alcohol abuse (p=0.006), (p=0.04) in women, but not in men.

**Conclusions:**

The study demonstrates for the first time that genetic predisposition to schizophrenia and mood disorders contributes to the simultaneous development of addictions in patients with schizophrenia, thereby worsening their quality of life. These results can help in developing strategies for prevention and cessation of tobacco and alcohol use. The study is supported by the Ministry of Science and Higher Education of the Russian Federation (the Federal Scientific-technical programme for genetic technologies development for 2019–2030, agreement No 075-15-2025-474 of 29.05.2025).

**Disclosure of Interest:**

None Declared

